# Provider attitudes about childbearing and knowledge of safer conception at two HIV clinics in Malawi

**DOI:** 10.1186/s12978-015-0004-0

**Published:** 2015-03-07

**Authors:** Paul Kawale, Deborah Mindry, Ann Phoya, Perry Jansen, Risa M Hoffman

**Affiliations:** Public Health Department, Nkhoma Hospital, Nkhoma, Malawi; Center for Culture and Health, University of California, Los Angeles, CA USA; Sector Wide Approach Secretariat, Ministry of Health, Lilongwe, Malawi; Partners In Hope Medical Center, Lilongwe, Malawi; David Geffen School of Medicine at the University of California, Los Angeles, Division of Infectious Diseases, Los Angeles, California USA; Private Bag 206, Lilongwe, Malawi

**Keywords:** HIV/AIDS, Reproductive health, Safer conception, Fertility desires

## Abstract

**Background:**

There is limited understanding of health care providers’ attitudes towards HIV-infected individuals’ reproductive choices, as well as knowledge about safer conception. Our study objective was to explore provider-level factors that serve as barriers and/or facilitators to the provision of reproductive and safer conception services for men and women living with HIV.

**Methods:**

Twenty-five providers were interviewed in four focus group discussions about their attitudes regarding childbearing by HIV-infected clients, reproductive health and HIV knowledge, and views and knowledge of safer conception.

**Results:**

Providers reported ambivalence about supporting childbearing among their clients with HIV. They raised concerns about HIV-infected individuals having children, and in certain cases expressed judgment that people with HIV should not have children because of these concerns. Providers lack specific knowledge about safer conception strategies and have low level of knowledge of reproductive health, the efficacy of PMTCT, and the risks of pregnancy for HIV-infected women.

**Conclusions:**

Providers in our setting have complex attitudes about HIV-infected clients having children and lack knowledge to appropriately counsel clients about reproductive health and safer conception. Our findings highlight need for further research in this area as well as the need for provider training in reproductive health and safer conception.

## Introduction

HIV-infected men and women need preconception guidance and counselling from healthcare providers in order to reduce transmission to seronegative partners and to reduce risk of transmission from mother-to-child [1-3]. Open discussion regarding sexual health and reproductive plans has become increasingly relevant in the context of HIV care given the large number of HIV-infected individuals of reproductive age, access to antiretroviral therapy (ART) ensuring a near normal lifespan, and a strong desire for children among HIV-infected men and women [4-9]. When clinicians are aware of clients’ reproductive desires, they can assist in providing or referring for preconception counselling, which may include: (1) improving health before conception, (2) identifying risk factors for adverse maternal or fetal outcomes and intervening to optimize outcomes, (3) ensuring a reliable contraception method until ready to attempt conception and re-initiation of contraception postpartum, and (4) preventing transmission of HIV to infants as well as sexual partners [2,10]. Preconception care across the reproductive lifespan is a critical component of comprehensive primary care for HIV-infected men and women.

Providers play a crucial role in determining access to, and quality of, reproductive health services [11] and influence childbearing decision-making among HIV-infected men and women [12,13]. However, there is limited understanding of health care providers’ knowledge and attitudes towards HIV-infected individuals’ fertility desires and reproductive choices [14,15], particularly in resource-limited settings [16]. We recently described fertility desires and intentions at two clinics in central Malawi showing that 51.0% of HIV-infected clients desired children. In-depth interviews with women revealed that 86% (n = 36 of 42 respondents) reported no discussion or a discouraging discussion with a provider about having children [17]. We therefore sought to explore provider-level factors that serve as barriers and/or facilitators to the provision of reproductive and safer conception services for men and women living with HIV at these same two clinics in central Malawi.

## Methods

This qualitative study was carried out in Malawi at a free HIV clinic (urban site), and at a large referral hospital that provides free HIV, antenatal, and maternity care (rural site), both in Lilongwe district. Data was collected in September and October, 2011. Twenty-five providers were recruited via announcements at staff meetings describing the study and requesting participants. We held two separate focus group discussions (FGDs) in English by trained local research assistants with twelve higher-level trained providers that included clinical officers, medical assistants, and nurses; and an additional two FGDs with thirteen lower-level trained providers that included HIV test counsellors, pharmacy workers, patient attendants, a laboratory technician and a ward clerk. Table 1 shows the number and type of participants interviewed in each of the four focus groups. FGDs lasted an average of one hour and no compensation was given for participation. After obtaining oral informed consent, providers were asked about their perceptions of clients’ fertility desires as well as barriers and facilitators to the provision of effective reproductive health services at their facility with specific attention to knowledge and views on safer conception methods for people living with HIV (PLHIV). Table 2 shows key questions from the FGD guide.

**Table 1:** **Health care providers interviewed in each of four focus group discussion groups**

	**Rural site**	**Urban site**	**Total**	
Clinical officers	1	3	**4**	Higher-level providers (N = 12)
Medical assistants	4	0	**4**	
Nurses	2	2	**4**	
HIV test counsellors	3	0	**3**	Lower-level providers (N = 13)
Patient attendant	1	4	**5**	
Lab technician	1	0	**1**	
Ward clerk	1	0	**1**	
Pharmacy assistant	0	3	**3**	
**Total**	**13**	**12**	**25**	

Groups were divided into providers with higher-level training and those with lower-level training.

**Table 2:** **Key questions from the focus group discussion guide**

**Provider attitudes**	**Childbearing among clients living with HIV**	What do you say to your HIV clients when they say that they would like to have children or that they are concerned about having children? Do you ask your clients about their desires and/or concerns about having children? How often? What have you heard or do you know about the attitudes or opinions of other health care providers regarding HIV clients’ desires to have children either at your clinic or in your experiences outside this clinic?
	**Partner involvement in decisions about having children**	Do you think health care providers should involve clients’ partners in the decision making process about having children and in the delivery of reproductive/family planning services? What are the challenges in Malawi for involving partners in reproductive health/family planning?
**Provider knowledge**	**HIV, reproductive health and PMTCT**	Have you had any training that has taught you about reproductive health/family planning needs of HIV clients? If so, in what way were you taught about meeting these needs?
	**Safer conception**	What do you tell clients about having a child when the female partner is HIV+ and the male partner is HIV negative? Is there any specific advice you give about how to prevent transmission to the HIV uninfected partner in this situation? What do you tell clients about having a child when the male partner is HIV+ and the female partner is HIV-negative? Is there any specific advice you give about how to prevent transmission to the HIV uninfected partner in this situation? What do you tell clients if both partners are HIV+ and want to have a child?

All of the sessions were recorded and transcribed. Three researchers reviewed the transcriptions and used open coding to identify preliminary themes. Qualitative data were coded with Atlas.ti (version 6.2, Berlin, Germany) using a grounded approach [18]. Themes developed for the interview guides shaped the dominant themes that emerged in the coding process. The open ended questions posed in the FGDs also enabled new themes and subthemes to emerge in the grounded approach to coding. After several re-readings, higher order concepts were developed and are reflected in the results section of this manuscript. Both the Malawi National Health Sciences Research Committee and the University of California Los Angeles (UCLA) Institutional Review Board approved the study.

## Results

### Provider attitudes about childbearing

Providers acknowledged the need to ask about fertility desires and for reproductive and safer conception counselling for people with HIV. Discussions about childbearing were reported to be both provider- and client-initiated. A provider explained how fertility options typically arise in discussions with a client who has just been diagnosed with HIV:

*There are people who come and test positive even if they’re not expecting, whether men or women, they usually sometimes ask, ‘Now that I have tested positive for HIV, is there a chance for me to have children?’ …The questions come with someone worrying and we encourage them by telling them that this is possible provided they follow the counselling that they receive at the hospital. We tell them the choice is theirs because the hospital cannot force them [to not have children].* (Lower-level provider, rural site)

During counselling, providers discussed the importance of providing clients with information and education, but ultimately leaving the decision about having children to the client. One provider said:

*Firstly if the client understands very well the counselling before ARVs and of course the importance of having children and, at the same time, their wellbeing, at least they make a sound choice. Nobody is banned from having children but at the same time their wellbeing is the one [that] is most important as well as the unborn baby. So they are given the choices and they have to make choices out of those things.* (Higher-level provider, urban site)

Providers were particularly sympathetic towards young couples and reported encouraging them to have children and acknowledged the importance of children to couples who have not yet had a child, as *“… some of the clients, they will come just married and they’re in need of a child, so when they come … we do encourage them that it’s possible but they have to know their CD4 count first before they start”*. (Higher-level provider, urban site) Another provider echoed similar sentiments:

*Let’s imagine maybe it’s a new couple, they are newlyweds, and they want a child. There is no way I can tell them not to get pregnant. I will encourage them to come … check their viral load, check their health status and also maybe their socioeconomic status, and then we will tell them this is the right time when you can get pregnant. But telling them not to get pregnant? I don’t think it will work.* (Higher-level provider, urban site)

And yet another provider explained that *“Many women have the desire to have children because their marriages can fail if they don’t and so they try hard to have children”*. (Lower-level provider, rural site)

While several providers acknowledged the need to talk with clients about desires for children, and about the cultural importance of children and the need to support clients, at least one demonstrated concerns about bearing the responsibility of counselling on safer conception, expressing fear of clients having an undesirable outcome:

*The important thing is that we advise them and not tell them, because if we tell them, then anything goes wrong they blame us, so rather we advise them on good nutrition, how their health is, what they should do not to re-infect each other.* (Lower-level provider, rural site)

Another provider also expressed opinions that HIV-infected clients should not be having children, particularly among couples who already have children:

*We … tell them that if they already have children and still want to continue giving birth, the difficulties that they might face, make them see that with the way my status is, it’s better just to stay and look after the few children I already have.* (Lower-level provider, rural site)

Concerns were also raised when both partners were HIV-positive, with providers citing the challenges of taking care of orphans, asking *“If you are encouraging somebody who is HIV positive to have children, what happens when he dies? Who will take care of those kids?”* (Higher-level provider, urban site)

*The child is HIV-positive, the parents are also HIV-positive and are maybe dead and the child will be an orphan. So the child is an HIV-positive orphan, [and] this is what makes the country to be poor, by helping such people [to have children].* (Lower-level provider, rural site)

### Provider attitudes about partner involvement in reproductive health

Providers demonstrated a strong belief that partners should be included in conversations about childbearing and the importance of couples counselling around decisions about children:

*We should also be encouraging the HIV couples to be coming together at the same time at the clinic … When you do counselling to a couple I think they really understand what you are talking about instead [of] just talking to an individual and that client tells his partner or her partner. So even [when] the wife talks to the husband, the husband doesn’t understand.* (Higher-level provider, urban site)

Another provider further supported partner involvement to ensure healthier pregnancy outcomes:

*Like maybe during delivery, we also need the partner to be there for assistance. There are risks of transmission of HIV to the baby so the partner needs to be involved so that they assist each other for the care of the pregnancy as well as the care of the baby. They will need family planning so we have to involve the partner.* (Higher level provider, urban site)

Providers expressed a strong sense of urgency in reaching out to people in rural communities to include partners in childbearing conversations.

*We medical personnel who have this information need to go out there and sensitize people … There should also be sensitization in villages with the chiefs to let them know the importance of both partners being present [for counselling about having children when one or both partners are HIV-positive].* (Lower-level provider, rural site)

### Provider knowledge of prevention of mother-to-child transmission (PMTCT) and safer conception strategies

Interviews with providers revealed limited knowledge about HIV and reproductive health, as well as PMTCT. One provider reported that HIV-infected individuals should not have children because of the risk of transmission and did not appear familiar with the effectiveness of antiretrovirals for PMTCT.

*Some get tired of using condoms and stop and have unprotected sex, which could lead to pregnancy, which could lead to having a child who would be HIV-positive, so we really encourage family planning.* (Lower-level provider, rural site)

Another provider explained his doubts about the effectiveness of antiretrovirals for PMTCT:

*We discourage against having children because of the risks involved, risks that the child can contract HIV are still there even if they’re taking medication, risks like infection when giving birth, and also some people still continue to breastfeed their babies even if they’re told not to because of poverty and this can lead to the child being infected with the virus and thereby adding problems to that child. So usually we counsel them to use condoms so that they do not get pregnant.* (Lower-level provider, rural site)

Providers also had mixed knowledge about the impact of pregnancy on maternal health and risks for complications such as pre-term labor. A provider expressed the following concern:

*A pregnant woman’s immunity normally drops so with the (HIV) virus it also drops further. This person can be in labor before their time since their immunity drops more.* (Lower-level provider, rural site)

Another provider concluded that,

*These people should be enlightened on the positives and negatives, the advantages and tell them the disadvantages… In the end, the choice is theirs that it’s not proper for them to have a child, like it has been explained concerning the immunity, which really goes down. So it’s not good to have a child since many times some die during childbirth.* (Lower-level provider, urban site)

Providers lacked specific knowledge about safer conception strategies. This was particularly notable when providers discussed couples with serodiscordant HIV status. One provider reported that they *“encourage them [HIV-serodiscordant clients] to continue using condoms so that the negative one should not contract HIV, they use condoms for the rest of their lives”*. (Lower-level provider, rural site), while another provider added:

*Okay, what we usually tell them is like we lay things on the table. You are HIV positive, you are HIV negative, [and] now you would like to have children. The idea is that if you would like to have children, … that means you will not be using the condoms, but the chances are that the partner who is not positive may end up having HIV.* (Higher-level provider, rural site)

Another provider also reported not providing any counselling about safer conception, but encouraged condom use, because *“the chances of getting the virus when you are involved in unprotected sex are high and it’s up to you to make a decision, but these are the risks. You can have a child, yes, but there are risks that are attached. So that is the message that is given when a man is negative and a woman is positive”*. (Higher-level provider, rural site)

One provider out of the 25 interviewed (4%) was able to describe a specific safer conception strategy:

*We would go using the same procedure we say that at least she has to go for viral load check, CD4 and then if the viral load is lower and the CD4 is higher, she is not sick she is healthy then she can be told when she will be fertile, of that ovulation time at least not to use condom for 3 to 4 days then to continue using condoms.* (Higher-level provider, urban site)

Providers were concerned about their limited knowledge of safer conception. Several providers mentioned that they send clients elsewhere for preconception counselling:

*When someone tests positive for HIV, there’s some counselling that is done. When that counselling is done, maybe that person can have more questions and if we fail to answer some questions, we refer that person to another section where they can be assisted appropriately on those particular issues.* (Lower-level provider, rural site)

## Discussion

### Providers have complex attitudes about HIV-infected clients having children

Health care providers in our focus groups were ambivalent about HIV-infected individuals having children. This has also been reported in Cape Town [5] and Ghana [19]. Providers believe in reproductive choices and recognize that they cannot make these decisions for their clients. Providers in our study are supportive of childbearing among their clients living with HIV, but support was largely reserved for certain situations including younger couples and people without children. Providers also acknowledge childbearing is a cultural norm and children play an important role in strengthening marriages. However, they raised concerns about risk to the mother’s health, risk to the infant’s health, and the social welfare of the child. Although providers in theory supported reproductive choice for their clients, on balance, providers in our study were more influenced by the potential risks of childbearing, leading them to discourage pregnancy, with few exceptions.

Certain service providers expressed an undertone of judgment related to people with HIV desiring children. Studies in Cape Town [15], Argentina [20], Nigeria [21,22] and Ghana [19] show that fertility choice among people with HIV is often not possible in a climate of judgmental and/or overtly negative attitudes of healthcare providers. In our previous study on fertility desires and intentions from these same two clinics, clients revealed the importance of provider authority and opinion in their decisions about childbearing [17]. There is a need to explore factors associated with negative provider attitudes, in order to alter the environment from one in which providers utilize their authority and personal beliefs to guide client decisions, to one that encourages a productive dialogue between providers and clients to facilitate supportive and non-coercive decision making around childbearing.

### Providers in our setting lack knowledge to appropriately counsel clients about safer conception

Providers trained in HIV care in these two clinics have very limited knowledge of reproductive health, PMTCT, and safer conception. This lack of knowledge may be contributing to the complex attitudes expressed towards HIV-infected clients having children. If providers lack experience with harm reduction strategies for safer conception, they may discourage behaviours they know to be associated with any risk of harm. Lack of knowledge has been shown to influence provider attitudes and opinions around supporting childbearing among people with HIV [23] and is further complicated by a lack of clear policy guidelines and training around safer conception counselling for HIV-infected individuals.

It is important to note that there are established safer conception strategies for HIV-infected clients in resource limited settings who desire children, including ART for the positive partner(s) [19,24], screening and treating for sexually transmitted infections, and timing unprotected sex with ovulation (male positive, female negative) or use of self-insemination (male negative, female positive) [25,26]. Bekker et al. have published clinical guidelines in this area for South Africa [27], but Malawi does not yet have a specific guideline or policy available to guide providers. Education of providers will not only enable them to deliver counselling to clients, but will provide information on harm reduction that can help providers feel comfortable that they are not putting clients (or future children) at high risk of an adverse outcome by offering these strategies. Providers’ enhanced understanding about topics such as the efficacy of ART for the prevention of MTCT, the efficacy of ART to prevent HIV transmission to partners, and the low risk of maternal harm during pregnancy when health status is optimized can help reduce provider fear and judgment, increase acceptance of clients having children, and facilitate providers to better support clients. Providers need to be educated and empowered to provide support for safer conception [14,28] because it is a health, legal, and human rights issue, as expressed in article 16 of the Universal Declarations of Human Rights: “*men and women of full age, without any limitation, have the right to marry and to found a family…”* [29].

### Providers recognize the importance of partner involvement in safer conception and reproductive health counselling

Providers in our study supported and encouraged partner disclosure and the inclusion of partners in counselling about reproductive health. Studies suggest that the participation of men in antenatal and delivery care is associated with better health seeking behaviour [30] and better reproductive health outcomes [31,32]. However, a prior study from Malawi reported providers did not believe men should be included in reproductive health care [33]. Kwambai et al. also describe negative attitudes of Kenyan providers to men’s participation in maternity care, which appeared to reinforce marginalisation of men and the notion that pregnancy care is a female domain [34]. The historic institutionalization of reproductive health as women’s health has generally resulted in health services that are not welcoming of men and has undermined efforts to engage couples [33-35]. Further, providers have limited knowledge of HIV-infected men’s reproductive intentions and have relative inexperience with male clients given the smaller number of men who seek treatment for HIV/AIDS [15,36]. There appears to be a complex interplay between provider and client attitudes and perspectives that make male partner involvement in reproductive health challenging. Additional studies are needed to identify factors that will facilitate male involvement, with a focus on systems issues related to health care facilities as well as barriers and facilitators at the provider, client, and partner level.

### Limitations

This was a small pilot study designed to gain preliminary data on provider attitudes in our program, and we did not have a large enough sample to differentiate results by site or to evaluate differences in provider attitudes in the urban versus rural setting. We did not perform sample size estimations and we did not collect demographic data on the providers who participated in the FGDs. The research design for this study was qualitative and our aim was not to characterize representativeness of ideas or to develop generalizable results, but rather to generate deeper insights into provider attitudes about childbearing in the context of HIV and their knowledge of safer conception methods. Our results require validation with a larger sample of providers and more rigorous methods to understand provider knowledge, such as in-depth or semi-structured individual interviews. Additionally, it is quite possible that in focus group discussions providers were more cautious about sharing personal views on PLHIV having children. Although we provided intensive training to interviewers, we acknowledge that this study may not reflect the full spectrum of views and attitudes among those interviewed.

## Conclusions

This study shows the complex perspectives of providers surrounding the issue of HIV-infected clients having children. It demonstrates the need for comprehensive training of providers in reproductive health and childbearing for PLHIV, with specific attention to safer conception methods and strategies to improve partner involvement. Such training should focus on addressing provider concerns about health risks to the mother and child and their concerns about the social welfare of the child, emphasizing how proper reproductive health, PMTCT, and safer conception counselling can minimize and potentially eliminate some of these risks. There is a need to develop policy and guidelines for healthcare providers in Malawi to better enable them to serve people with HIV intending to have children.
